# *Notice to Readers*: Change in Publication Date of *MMWR* Series

**DOI:** 10.15585/mmwr.mm7301a5

**Published:** 2024-01-11

Effective with this issue, the official publication date for the *MMWR* Series of publications (i.e., Recommendations and Reports, Surveillance Summaries, Supplements, and Weekly) will be Thursday instead of Friday to align with the Thursday embargo release. For the immediate release of important public health information, *MMWR* will continue to publish some reports outside the routine weekly publication schedule.

